# Comparative Analysis of Selected UGT and SULT mRNA Expression in Non-Obese Rat Models of Metabolic Syndrome

**DOI:** 10.3390/biomedicines14061206

**Published:** 2026-05-27

**Authors:** Jan Soukop, Martin Poruba, Zuzana Rácová, Iveta Zapletalová, Hana Malínská, Martina Hüttl, Irena Marková, Rostislav Večeřa

**Affiliations:** 1 Department of Pharmacology, Faculty of Medicine and Dentistry, Palacky University Olomouc, 779 00 Olomouc, Czech Republiciveta.zapletalova@upol.cz (I.Z.); 2 Centre for Experimental Medicine, Institute of Clinical and Experimental Medicine, 140 21 Prague, Czech Republic

**Keywords:** metabolic syndrome, phase II enzymes, drug metabolism, rat model

## Abstract

**Background:** Metabolic syndrome (MetS) is a cluster of risk factors increasing the likelihood of cardiovascular and metabolic diseases. **Objectives:** This study investigated the relative mRNA expression of key hepatic and intestinal phase II drug-metabolising enzymes, specifically UDP-glycosyltransferases (UGTs) and sulfotransferases (SULTs), in four non-obese rat models of MetS characterised by different dominant traits: the hereditary hypertriglyceridaemic (HHTg) rat, spontaneously hypertensive rat (SHR), SHR-expressing transgenic human C-reactive protein (SHR-CRP) rat, and bilaterally ovariectomised female Wistar (W-OVX) rat, compared to Wistar controls. **Methods:** Gene expression was quantified by RT-PCR with data normalised using the ΔΔCt method. **Results:** Measurements showed significant model-specific differences, especially in the liver. HHTg rats exhibited significant hepatic suppression of *Ugt1a9* (−90%) and undetectable *Ugt2b* transcripts, alongside compensatory upregulation of *Sult1a1* (196%) and *Sult1b1* (277%). The SHR model showed significant hepatic upregulation of *Ugt1a1* (330%), *Sult1a1* (266%), and *Sult1b1* (328%). Chronic inflammation in SHR-CRP rats caused a significant decrease in hepatic *Ugt1a1*, whereas a significant induction occurred in the intestine. Oestrogen deprivation (W-OVX) led to significant downregulation of hepatic *Ugt1a6* and *Ugt1a9*. **Conclusions:** These findings highlight that the alterations in phase II metabolism strongly depend on the pathophysiological context, potentially affecting drug disposition in preclinical models.

## 1. Introduction

Metabolic syndrome (MetS) represents a cluster of interrelated metabolic disorders characterised by hypertension, dyslipidaemia, insulin resistance and altered glucose homeostasis, collectively increasing the risk of cardiovascular and metabolic diseases [1]. Whilst obesity is commonly associated with MetS, non-obese phenotypes of metabolic dysfunction have garnered increasing attention as they represent a distinct pathophysiological entity that may reflect genetic predisposition or specific metabolic derangements [2].

Non-obese animal models of MetS offer valuable tools for dissecting the molecular mechanisms underlying these conditions whilst circumventing the confounding effects of adiposity. Our previous work examined cytochrome P450-mediated (phase I) drug metabolism across various non-obese rat models, revealing significant inter-strain differences in gene expression of key drug-metabolising enzymes and nuclear receptors [3]. Phase I metabolism, catalysed primarily by the CYP superfamily, is responsible for the initial modification of xenobiotics. However, the efficiency of drug clearance and the generation of metabolites often depends critically on phase II biotransformation enzymes, which catalyse conjugation reactions that enhance hydrophilicity and facilitate elimination [4].

The two major families of phase II enzymes are UDP-glycosyltransferases (UGTs) and sulfotransferases (SULTs) [4]. While the UGT superfamily encompasses enzymes that transfer various UDP-sugars, the specific UGT1 and UGT2 families analysed in this study primarily catalyse glucuronidation of endogenous substrates (e.g., oestrogens) and xenobiotics, whereas SULTs mediate sulphation of phenolic and hydroxylic compounds [4]. Both enzyme families display tissue-specific and sex-specific expression patterns. Their activity can be modulated by metabolic stress, systemic inflammation and various hormonal factors [5].

The pathophysiological hallmarks of MetS, particularly chronic low-grade inflammation, oxidative stress, and dyslipidaemia, impact both hepatic and extrahepatic tissues, directly affecting the expression and activity of drug-metabolising enzymes [6]. While the impact of MetS on CYP is well documented, emerging evidence highlights that phase II pathways are also highly susceptible to these metabolic disturbances. The dysregulation of nuclear receptors (PXR, CAR, AhR) driven by pro-inflammatory cytokines and altered lipid homeostasis can lead to significant shifts in UGT an SULT expression profiles [7]. Furthermore, because UGTs and SULTs are responsible not only for metabolism of xenobiotics, but also for the biotransformation of endogenous signalling molecules, their alteration in MetS can create a pathophysiological feedback loop that further exacerbates metabolic dysfunction [8,9].

To date, comprehensive comparative data on UGT and SULT mRNA expression across multiple non-obese MetS models remain limited. This article extends our previous findings by systematically examining hepatic and intestinal UGT and SULT mRNA expression in established rat models representing distinct phenotypes of MetS. The specific UGT and SULT genes analysed in this study were selected on the basis of their established pharmacological relevance, their documented expression in rat hepatic and intestinal tissues, and their shared transcriptional regulation by the nuclear receptors studied in our previous work. To dissect the individual impact of specific cardiometabolic stressors, we have selected four male strains, the hereditary hypertriglyceridaemic (HHTg) rat, the spontaneously hypertensive rat (SHR), the SHR with transgenic human C-reactive protein (SHR-CRP) and the Wistar rat as a control group. We also compared ovariectomised female Wistar (W-OVX) rats with SHAM-operated female Wistar (W-SHAM) rats to clarify the impact of postmenopausal oestrogen deprivation. Our aim is to determine how these specific pathophysiological components individually drive strain-specific and tissue-specific patterns of phase II enzyme expression.

## 2. Materials and Methods

The animal strains, experimental design, and ethical procedures were described in detail in our previous study, which was focused on selected phase I enzymes, ABC transporters and nuclear receptors [3]. The metabolic and physiological characteristics of the specific animal cohorts used in this study, including body weight, plasma lipid levels, blood pressure and glucose homeostasis markers, were also characterised in our previous work [3]. A detailed summary of phenotypic data is available in Table S1 of the aforementioned publication. Briefly, the study included four groups of 7–9-month-old male rats, namely Wistar rats serving as the control group, and HHTg, SHR, and SHR rats with transgenic human C-reactive protein (SHR-CRP). To investigate sex-specific differences, two groups of seven-month-old female Wistar rats were also included: SHAM-operated Wistar (W-SHAM) and ovariectomised Wistar (W-OVX) rats. All animals were maintained under standard housing conditions with free access to food and water. The study was performed in accordance with the guidelines and practices established by the Animal Care and Use Committee of the Institute for Clinical and Experimental Medicine (IKEM), Prague, which accord with the European Convention on Animal Protection and Guidelines on Research Animal Use, and were approved by this committee and subsequently by the Ministry of Health of the Czech Republic (the decision number for this project is 25976/2016-OVZ and 27513/2022-5/OVZ). All experiments are in accordance with the ARRIVE guidelines.

### 2.1. Tissue Preparation and Real-Time Quantitative RT-PCR

Following the protocol established in our previous study, all animals were euthanised by decapitation under light anaesthesia in a postprandial state [3]. Samples of the liver and small intestine were immediately washed in saline and stored at −80 °C. Total RNA was isolated from the samples using the RNeasy Mini Kit (Qiagen, Hilden, Germany) according to the manufacturer’s instructions. RNA quality and concentration were determined spectrophotometrically, ensuring the criteria were met as previously described [3]. RNA purity was confirmed by A260/280 and A260/230 ratios ranging between 1.8 and 2.1. To ensure robust normalisation, *Hprt1* was utilised as the endogenous reference gene, having demonstrated stable expression across all experimental groups in both hepatic and intestinal tissues. A transcript was considered non-detectable if the quantification cycle (Cp) values exceeded 40 cycles. Complete information regarding primer sequences is provided in Supplementary Table S1. Commercially available, pre-validated primer assays were utilised, which guarantee optimal amplification efficiency for all targets. To extend the initial analysis of xenobiotic metabolism, we assessed the mRNA expression of key UGTs and SULTs. The obtained data were normalised using the ΔΔCt method, and the values of the control groups were set to 100%.

### 2.2. Statistical Analysis

Statistical analysis was performed with TIBCO Statistica software (DataBon, Prague, Czech Republic, ver. 14.0.0.15). Prior to parametric testing, data were evaluated for normal distribution using the Shapiro–Wilk test. While some variables exhibited deviations from strict normality, parametric tests were utilised due to their established robustness when group sample sizes are balanced. One-way ANOVA with a subsequent post hoc Bonferroni correction was used for the comparison of the Wistar, HHTg and SHR groups. For the comparison of the groups (SHR and SHR-CRP, W-SHAM and W-OVX), an unpaired Student’s *t*-test was used. Statistical significance was defined as *p* < 0.05.

## 3. Results

### 3.1. Comparison of Hepatic UGT and SULT mRNA Expression in Wistar, HHTg and SHR Rats

**Figure 1:**
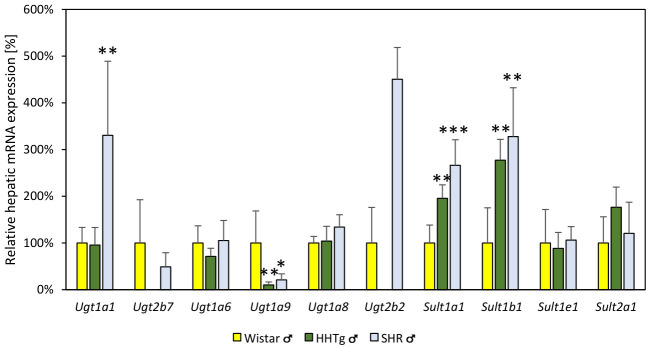
Differences in the relative mRNA expression of UGT and SULT isoforms in the liver tissues of male HHTg and SHR rats compared with control male Wistar rats, with their values set to 100%. Data are expressed as the mean ± SEM, n = 5–6. * Denotes *p* < 0.05; ** denotes *p* < 0.01; *** denotes *p* < 0.001 compared with the Wistar group.

Differences in the relative mRNA expression of selected UGT and SULT isoforms in the hepatic tissue of HHTg and SHR rats, compared with the control Wistar strain, are illustrated in Figure 1. HHTg rats exhibited a statistically significant reduction in *Ugt1a9* expression to 10% compared to Wistar rats (*p* < 0.05). Conversely, a significantly increased expression to 196% (*p* < 0.01) for *Sult1a1* and to 277% (*p* < 0.01) for *Sult1b1* was observed. Notably, mRNA expression for *Ugt2b7* and *Ugt2b2* was not detected in the HHTg strain. The remaining isoforms analysed in this strain showed no statistically significant differences relative to controls. As for the SHR group, a statistically significant reduction in Ugt1a9 expression to 21% (*p* < 0.01) was also observed in comparison with the Wistar group. In contrast to the Wistar controls, SHR rats demonstrated highly statistically significant increases to 330% (*p* < 0.01) in the expression of Ugt1a1, to 266% (*p* < 0.001) for Sult1a1, and to 328% (*p* < 0.01) for Sult1b1. Regarding the other isoforms in the SHR strain, the mean expression of *Ugt2b2* reached 451% of the control value; however, this difference did not achieve statistical significance. The expression levels of the remaining isoforms analysed in SHR rats did not differ significantly from the Wistar group.

### 3.2. Effect of Human C-Reactive Protein on Hepatic UGT and SULT mRNA Expression in SHR Rats

**Figure 2:**
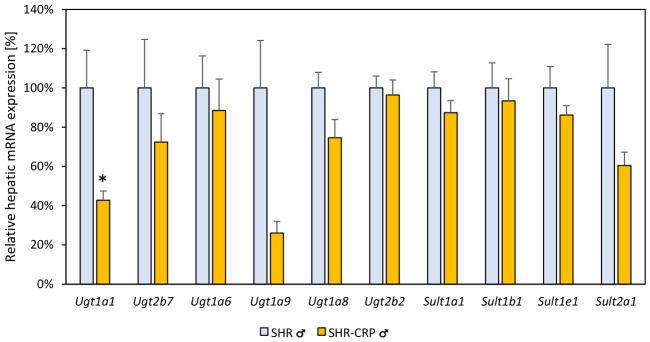
Differences in the relative mRNA expression of UGT and SULT isoforms in the liver tissues of male SHR-CRP rats compared with male SHR rats, with their values set to 100%. Data are expressed as the mean ± SEM, n = 5–6. * Denotes *p* < 0.05 compared with the SHR group.

Differences in the relative mRNA expression of selected UGT and SULT isoforms in the hepatic tissue of male SHR-CRP rats, compared with the control male SHR rats, are demonstrated in Figure 2. Compared with the control SHR group (set as the 100% baseline), the SHR-CRP rats exhibited a statistically significant reduction in *Ugt1a1* expression to approximately 43% (*p* < 0.05). A trend towards decreased mean expression was also observed for all other isoforms analysed. However, these differences did not achieve statistical significance. The most pronounced, though statistically non-significant, reductions to approximately 26% for *Ugt1a9* and to approximately 60% for Sult2a1 were noted.

### 3.3. Effect of Ovariectomy on Hepatic UGT and SULT mRNA Expression in Female Wistar Rats

**Figure 3:**
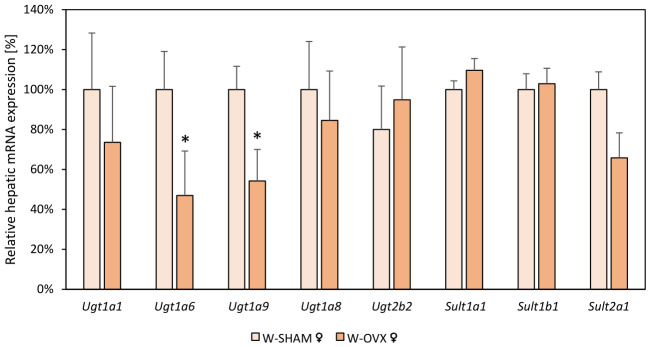
Differences in relative mRNA expression of UGT and SULT isoforms in the liver tissues of ovariectomised female Wistar (W-OVX) rats compared to SHAM-operated control (W-SHAM) rats, with their values set to 100%. Data are expressed as the mean ± SEM, n = 5. * Denotes *p* < 0.05 compared with the W-SHAM group.

The effect of ovariectomy on the relative mRNA expression of hepatic UGT and SULT isoforms in female Wistar (W-OVX) rats is shown in Figure 3. The baseline for the control group, SHAM-operated female Wistar (W-SHAM) rats, was set to 100%. The W-OVX group exhibited statistically significant downregulation of *Ugt1a6* to 47% and of *Ugt1a9* to 54% in comparison with the W-SHAM group (*p* < 0.05). A non-significant trend towards reduced expression in the W-OVX group was also observed in *Ugt1a1*, *Ugt1a8* and *Sult2a1*. The expression levels of the remaining isoforms analysed did not differ significantly between the W-OVX and W-SHAM groups.

### 3.4. Comparison of Intestinal UGT and SULT mRNA Expression in Wistar, HHTg and SHR Rats

**Figure 4:**
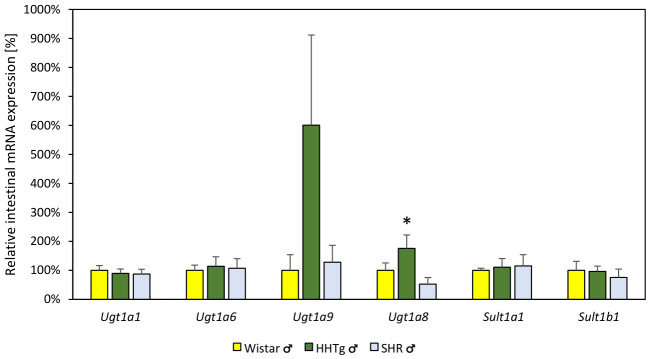
Differences in the relative mRNA expression of UGT and SULT isoforms in the small intestine tissues of male HHTg and SHR rats compared with male Wistar rats, with their values set to 100%. Data are expressed as the mean ± SEM, n = 5–7. * Denotes *p* < 0.05 compared with the Wistar group.

The differences in relative mRNA expression of selected UGT and SULT isoforms in the small intestine tissues of male HHTg and SHR rats, compared with control male Wistar rats, are presented in Figure 4. In the HHTg strain, a statistically significant increase in *Ugt1a8* expression was observed, reaching approximately 176% of the Wistar control (*p* < 0.05). A substantial, though statistically non-significant, mean increase was also noted for *Ugt1a9* in this group, to approximately 600%, but this finding was associated with high biological variability, as indicated by the SEM. No statistically significant differences from the Wistar controls were found for any of the other isoforms analysed in the HHTg strain, nor for any of the isoforms measured in the SHR strain.

### 3.5. Effect of Human C-Reactive Protein on Intestinal UGT and SULT mRNA Expression in SHR Rats

**Figure 5:**
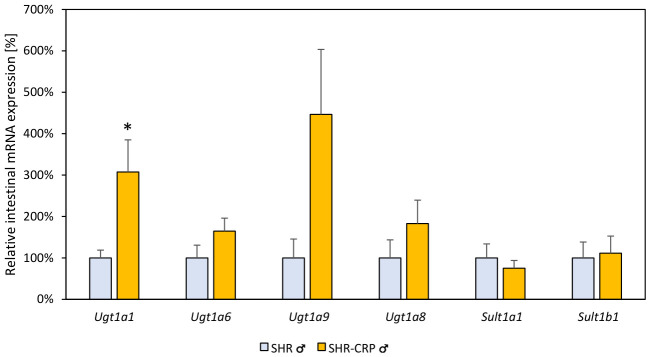
Differences in the relative mRNA expression of UGT and SULT isoforms in the small intestine tissues of male SHR-CRP rats compared with male SHR rats, with their values set to 100%. Data are expressed as the mean ± SEM, n = 5–6. * Denotes *p* < 0.05 compared with the SHR group.

The effect of human C-reactive protein on the relative mRNA expression of selected UGT and SULT isoforms in the small intestine tissues of male SHR-CRP rats, compared with control male SHR rats, is shown in Figure 5. The SHR-CRP group demonstrated a statistically significant increase in *Ugt1a1* expression, which reached 307% of the control SHR group (*p* < 0.05). Although mean expression levels for *Ugt1a6*, *Ugt1a9* and *Ugt1a8* were also elevated in the SHR-CRP group, these changes did not achieve statistical significance. The substantial mean increase observed for *Ugt1a9* was associated with high data variability. The expression levels of *Sult1a1* and *Sult1b1* did not differ significantly between the two groups.

### 3.6. Effect of Ovariectomy on Intestinal UGT and SULT mRNA Expression in Female Wistar Rats

**Figure 6:**
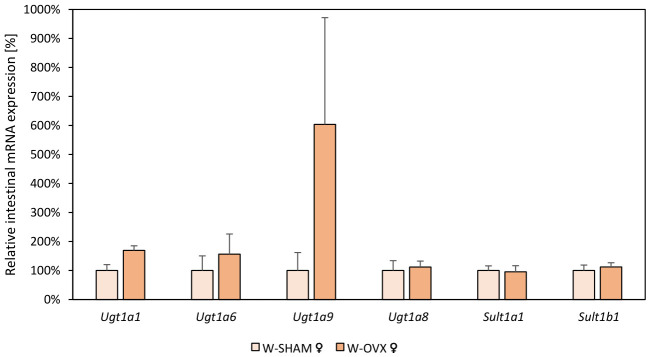
Differences in the relative mRNA expression of UGT and SULT isoforms in the small intestine tissues of female W-OVX Wistar rats compared to female SHAM-operated Wistar rats, with their values set to 100%. Data are expressed as the mean ± SEM, n = 5.

The effect of ovariectomy on the relative mRNA expression of UGT and SULT isoforms in the small intestine of female Wistar (W-OVX) rats, compared with SHAM-operated control (W-SHAM) rats, is presented in Figure 6. No statistically significant differences were found between the W-OVX and W-SHAM groups for any of the isoforms analysed. However, a substantial mean increase in *Ugt1a9* expression was observed in the W-OVX group, reaching approximately 604% of the SHAM control group. This pronounced change did not achieve statistical significance, as it was associated with extremely high biological variability within the group, as indicated by the large SEM. Non-significant trends towards increased expression in the W-OVX group were also noted for *Ugt1a1* and *Ugt1a6*. The expression levels of *Ugt1a8*, *Sult1a1*, and *Sult1b1* remained largely unchanged compared with the W-SHAM group.

## 4. Discussion

Glycosylation and sulfation represent the key mechanisms for the elimination of xenobiotics and the regulation of endogenous signalling molecules, including bile acids and steroids [10,11]. Under physiological conditions, the phase II enzymes effectively maintain metabolic homeostasis and prevent toxicity. In contrast, our data indicate that under pathological conditions, such as hypertriglyceridaemia, hypertension, and ovariectomy-induced menopause, the transcriptional regulation pathways related to the metabolic capacity may be altered, suggesting a potential compromise.

### 4.1. The mRNA Expression of UGT and SULT Enzymes in Hypertriglyceridaemic State

The HHTg rat model replicates the prevalent condition of human hypertriglyceridaemia and the metabolic disturbances associated with it [12]. We previously reported that despite the hypertriglyceridaemic state, cholesterol levels in HHTg rats remained comparable to controls, an effect that was attributed to a compensatory balance between a downward trend in hepatic *Abcg5/Abcg8* expression and an upward trend in *Cyp7a1* [3].

Our current investigation into phase II metabolism reinforces this theme of hepatic compensation. As seen in Figure 1, the primary finding in the HHTg liver was the significant suppression of hepatic *Ugt1a9* expression. This is highly consistent with our previous report of a downward trend in its key transcriptional factors, PXR/*Nr1i2* and *Ahr*, and the significant suppression of another key target, *Cyp1a2* [3]. This concurrent suppression of phase I (*Cyp1a2*) and phase II (*Ugt1a9*) enzymes, both regulated by the same suppressed nuclear receptors, indicates a broad, transcriptionally driven impairment of the liver’s capacity to metabolise xenobiotics and specific endogenous substrates [13]. This hepatic downregulation appears to trigger distinct compensatory mechanisms. We also observed a significant hepatic upregulation of the sulfotransferase isoforms *Sult1a1* and *Sult1b1*. The parallel suppression of phase I and phase II enzymes regulated by the same transcriptional pathways strongly suggests a coordinated, transcriptionally driven impairment of selected hepatic detoxification routes in the hypertriglyceridaemic state [13,14].

Members of the UGT2B subfamily contribute substantially to the glucuronidation of bile acids and steroid hormones, thereby increasing their hydrophilicity and facilitating biliary and urinary excretion in mammals [15]. The non-detectable expression of *Ugt2b* isoforms in HHTg livers suggests a possible transcriptional deficit that may translate to reduced hepatic capacity for glucuronidation of these key endogenous substrates, which may contribute to altered bile acid and sterol handling under dyslipidaemic conditions [16].

When interpreted alongside our prior observations of altered *Cyp7a1* expression and suppressed sterol transporters *Abcg5* and *Abcg8*, these data collectively suggest a potential impairment of hepatic bile acid and sterol conjugation capacity in HHTg rats, rather than a complete failure of cholesterol metabolism [3]. Conjugation reactions mediated by UGT and SULT enzymes represent major phase II detoxification pathways that integrate both xenobiotic and endobiotic metabolic fluxes through transcriptional control by nuclear receptors such as PXR, CAR and PPARs [17]. Thus, the observed *Ugt2b* deficiency may reflect a transcriptionally mediated shift in hepatic detoxification programming.

Interestingly, this apparent suppression of glucuronidation pathways was accompanied by a significant upregulation of the sulfotransferases *Sult1a1* and *Sult1b1* in the liver. Phase II enzyme families exhibit overlapping substrate specificity for many phenolic compounds, steroids and bile acids, and both sulfation and glucuronidation often act as complementary conjugation pathways for the same substrates, with their relative contribution changing in response to metabolic or inflammatory stress [18,19]. While functional validation is required, the observed induction of SULTs is consistent with an adaptive response that helps maintain overall conjugation capacity within the liver when specific high-capacity glucuronidation routes are compromised [13,18].

In contrast to the liver, the intestinal tissue of HHTg rats exhibited an opposing regulatory pattern. We observed a significant induction of *Ugt1a8* and a pronounced, albeit highly variable, increase in *Ugt1a9* expression. This finding closely mirrors our previous report of intestinal upregulation of the nuclear receptors AhR, PXR/Nr1i2 and CAR/Nr1i3 in this strain [3]. The concordant induction of transcription factors and their downstream phase II targets strongly supports the concept of a transcriptionally driven enhancement in intestinal metabolic capacity. Enhanced intestinal phase II activity may therefore serve as an extrahepatic compensatory mechanism, buffering systemic exposure to lipophilic endogenous substrates and xenobiotics when hepatic detoxification is compromised [20,21].

Taken together, these findings define a distinctive metabolic fingerprint of the HHTg model characterised by hepatic suppression of selected PXR/AhR-regulated phase I and phase II pathways, combined with compensatory induction of sulfation in the liver and enhanced glucuronidation capacity in the intestine. This liver–intestine contrast highlights the importance of tissue-specific adaptive responses in hypertriglyceridaemia and suggests that extrahepatic metabolism may partially compensate for altered hepatic conjugation under dyslipidaemic conditions.

### 4.2. The mRNA Expression of UGT and SULT Enzymes in Hypertensive State

While the HHTg model is primarily defined by dyslipidaemia, the SHR rat serves as a more direct and established model for human essential hypertension [22,23]. In our previous work, we identified a unique hepatic fingerprint in SHR rats, notably the significant upregulation of *Cyp3a23* (human CYP3A4 orthologue) and *Cyp2d1* (human CYP2D6 orthologue), alongside the downregulation of *Cyp1a2* and *Cyp2a1* [3]. Our new data on phase II enzymes not only reinforce this unique SHR phenotype but also significantly expand upon it. The suppression of hepatic *Cyp1a2*, which we previously linked to a downward trend in hepatic *Pxr*, is now mirrored by a significant downregulation of hepatic *Ugt1a9*. This concurrent suppression of both a key phase I and phase II enzymes, both of which are known PXR/AhR targets, strengthens the hypothesis that a broad transcriptional suppression of this specific metabolic pathway is a characteristic of the SHR liver [24].

However, the most compelling findings relate to the specific inductions observed in this model. Our previous report of upregulated *Cyp3a23*, *Cyp2d1*, and *Cyp2c6* is now complemented by the highly significant upregulation of *Ugt1a1* and the SULT isoforms *Sult1a1* and *Sult1b1*. This is a critical finding, as the induction of *Ugt1a1* is unique to the SHR strain and was not observed in HHTg rats. This collective upregulation of multiple, mechanistically distinct phase I and phase II enzymes (CYPs, UGTs, and SULTs) suggests a complex hepatic adaptation to the hypertensive state, far different from the simple suppression seen with inflammation [3,25]. Furthermore, the UGT expression data clearly distinguish between our two pathological models. Unlike HHTg rats, which exhibited absent *Ugt2b7/2b2* transcripts, SHR maintained normal, Wistar-level expression of these isoforms, indicating preserved bile acid conjugation capacity and distinguishing hypertension pathophysiology from HHTg dyslipidaemia. In the small intestine, the SHR strain did not show any significant alterations in any measured UGT or SULT isoforms. This stands in contrast to the HHTg model, where we observed significant intestinal PXR/CAR induction and a corresponding upregulation of UGTs. This expanded enzymatic profile presents potential clinical implications, although highly hypothetical without further validation. As we noted previously, almost 80% of individuals with MetS suffer from hypertension and are at risk of drug–drug interactions [1]. Our refined data show that the significant upregulation of *Cyp3a23*, *Cyp2d1*, *Cyp2c6*, and now *Ugt1a1*, *Sult1a1*, and *Sult1b1*, coupled with the downregulation of *Cyp1a2* and *Ugt1a9*, could theoretically alter the metabolism of drugs used for treating MetS disorders [3].

This altered enzymatic distribution suggests the potential to affect the pharmacokinetics of common therapies, such as antiarrhythmics or β-blockers, underscoring the need for careful therapeutic management in hypertensive patients.

### 4.3. The mRNA Expression of UGT and SULT Enzymes in a Condition of Chronic Inflammation

Metabolic syndrome is linked to a state of chronic low-grade inflammation, with C-reactive protein serving as a key biomarker [26]. As detailed in our previous work, we used the SHR-CRP model to investigate the effects of human CRP, which is known to be pro-inflammatory in rats. Our new data on phase II enzymes provide crucial functional context to our earlier findings on nuclear receptors, revealing a striking, tissue-specific response to inflammation.

In the liver, our findings align with the established pattern of inflammation-mediated suppression of hepatic metabolism. We observed a significant downregulation of hepatic *Ugt1a1*, alongside a general non-significant downward trend for most other measured UGT and SULT isoforms. This suppression of phase II enzymes complements our previous data on CYPs and the broader literature, which indicates that CRP negatively modulates hepatic gene expression [3,27,28]. Interestingly, this suppression occurs despite our previous observation of a significant upregulation of *Nr1i3* in the SHR-CRP liver. This suggests that the pro-inflammatory signalling cascade is dominant, effectively overriding the transcriptional activity of CAR and repressing target gene expression, a well-documented phenomenon during the acute-phase response [27,28].

In contrast, the response in the small intestine was diametrically opposed and provides a functional validation of our previous hypothesis. We measured a significant, threefold induction of intestinal *Ugt1a1*, along with strong upward trends for *Ugt1a9* and Ugt1a8. In our previous study, we reported a significant upregulation of the intestinal nuclear receptors *PXR/Nr1i2* and *CAR/Nr1i3* in these same animals, and we hypothesised this would impact intestinal metabolism [3]. The *Ugt1a1* induction we now report provides evidence that the CRP-driven inflammation induces intestinal nuclear receptors, which in turn upregulate the expression of their target Phase II enzymes.

Taken together, the current findings reveal a complex, tissue-specific role for chronic inflammation. CRP appears to mediate a dual response, a systemic suppression of hepatic metabolism (diminishing clearance of drugs via *Ugt1a1*) while simultaneously “fortifying the gut barrier” via a PXR/CAR-mediated induction of intestinal phase II enzymes (*Ugt1a1*). This intestinal response may serve as a critical defence mechanism, enhancing the gut’s metabolic capacity to limit the systemic translocation of microbial products or other pro-inflammatory stimuli from the lumen [29,30,31].

### 4.4. The mRNA Expression of UGT and SULT Enzymes in Postmenopausal State

The increased prevalence of metabolic syndrome in postmenopausal women is a significant clinical issue, a state we investigated using the ovariectomised (W-OVX) rat model [32,33]. Our previous analysis in these animals revealed a specific hepatic downregulation of *Gsta1* and intestinal upregulation of *Abcg5* and *Abcb1* [3]. Our new data on phase II enzymes further support the tissue-specific nature of this hormonal regulation and also provide critical mechanistic links to the transcriptional pathways we previously identified [3].

In the liver, we observed a significant downregulation of both *Ugt1a6* and *Ugt1a9* (Figure 3). The suppression of *Ugt1a9* is particularly noteworthy. It strongly complements our previous observation of a downward trend in both its key regulator, AhR, and the well-known phase I enzyme *Cyp1a2*. This concurrent, hormone-driven suppression of both a phase I (*Cyp1a2*) and a phase II (*Ugt1a9*) enzyme suggests a coordinated downregulation of the AhR-mediated metabolic pathway in response to oestrogen loss [3,34]. This finding is also consistent with human data showing decreased CYP1A2 activity in postmenopausal women [34]. Interestingly, this suppression appears highly specific, as other hepatic pathways, such as those represented by *Ugt1a8*, *Sult1a1*, and *Sult1b1*, remained unaffected.

As seen in other models in this study, the intestinal expression in W-OVX rats was also different from that of the liver. In our previous study, we reported significant intestinal upregulation of the ABC transporters *Abcg5* and *Abcb1* [3]. Our current data reinforce this finding. We observed a substantial, albeit statistically non-significant, mean increase of approximately 600% in intestinal *Ugt1a9*, along with upward trends for *Ugt1a1* and *Ugt1a6*.

Although the high inter-individual variability, likely due to sampling challenges as discussed previously, prevented this *Ugt1a9* change from reaching statistical significance, its direction and sheer magnitude are perfectly aligned with the significant inductions of *Abcg5* and *Abcb1* we previously reported, as well as the upward trends in *Cyp1a1* and *Cyp2c6* [3]. Taken together, these data strongly suggest that oestrogen deprivation triggers a broad transcriptional upregulation of the intestinal metabolic barrier, an effect that is diametrically opposed to the specific suppression observed in the liver [35].

### 4.5. Limitations of the Study

The conclusions drawn from this study on phase II UGT and SULT enzymes are subject to several limitations, many of which are shared with our previous work on phase I enzymes, transporters and transcription factors.

Firstly, the selected rat models successfully replicate specific features of human MetS, but it is important to recognise that they do not fully capture the complete clinical and pathophysiological complexity observed in humans. Each model provides valuable insight into isolated aspects of the condition: HHTg rats primarily model dyslipidaemia, SHR rats reflect hypertension, W-OVX rats simulate oestrogen deficiency, and the SHR-CRP model introduces a component of low-grade inflammation. However, human MetS is multifactorial and polygenic, involving a complex interaction of metabolic disturbances that are not comprehensively represented across these individual animal models. This inherent difference poses significant challenges for direct application of our preclinical findings. For instance, the HHTg model, while an excellent model of HHTg, has limitations in reflecting the full spectrum of human dyslipidaemia, which must be considered when interpreting our UGT findings related to bile acid metabolism. Similarly, the SHR-CRP model’s restricted metabolic profile, lacking broader disturbances common in human inflammatory MetS, must be considered when interpreting the inflammation-driven changes in UGT expression.

Secondly, while metabolic syndrome is inherently associated with oxidative stress and potential alterations in glutathione (GSH) status, this study focused exclusively on UGT and SULT pathways. GSH conjugation, specifically the transcript expression of glutathione S-transferase alpha 1 (*Gsta1*), was evaluated in our previous study on these exact animal cohorts [3]. To logically extend those findings and avoid data duplication, the current work prioritised the UGT and SULT superfamilies due to their shared transcriptional regulation by the nuclear receptors analysed in our phase I study [3]. Nevertheless, the exclusion of a broader panel of GST isoforms and direct functional GSH assays in the present manuscript represents a limitation when evaluating the comprehensive antioxidant and detoxification capacity of these models.

Thirdly, our experimental design relied on established genetic and transgenic models, which differ fundamentally from diet-induced models of MetS. Diet-induced rodent models, typically generated via high-fat, high-carbohydrate, or high-fructose diets, are highly informative as they closely replicate the nutritional aetiology and multifactorial pathogenesis of human MetS [36]. However, diet-induced models frequently develop obesity and can exhibit significant phenotypic variability, making it difficult to uncouple the effects of specific MetS components from generalised adiposity. In contrast, the genetic and transgenic models utilised in our study offer the ability to isolate specific, dominant cardiometabolic stressors strictly within a non-obese context. This allows for a more targeted mechanistic dissection of how individual pathological factors modulate phase II enzymes. Nevertheless, the lack of a diet-induced pathogenesis in our models represents a limitation, as they may not fully capture the complete metabolic complexity of human MetS.

A principal limitation of this study, as in our previous work, is its reliance on mRNA expression analysis. The observed changes in UGT and SULT transcripts, while substantial, do not always directly correlate with protein levels or, more critically, functional enzyme activity. For example, the non-detectable *Ugt2b* transcripts in HHTg rats are a strong finding, but confirmation at the protein level is essential. Therefore, verification of these findings using proteomics and functional assays is crucial to confirm their physiological relevance.

Furthermore, several key findings, particularly in the small intestine (e.g., *Ugt1a9* in HHTg and W-OVX rats), were characterised by extremely high inter-individual variability. This variability, while likely biological, prevented substantial mean changes from reaching statistical significance, as our study utilised a relatively small sample size for each experimental group (n = 5–6).

A statistical limitation relates to the multiple comparisons across numerous genes. As this study was designed as an exploratory investigation, we treated the expression of each individual UGT and SULT isoform as an independent hypothesis. Therefore, we did not apply a global statistical adjustment (such as the False Discovery Rate) across all tested transcripts, as this could increase type II errors and obscure potentially relevant biological trends. Consequently, we cannot entirely exclude the possibility of false-positive findings, and the statistically significant alterations reported herein should be considered preliminary targets requiring future functional validation. Furthermore, when translating preclinical findings to human pharmacology in general, the interspecies differences must be considered. Although rats and humans share functional orthologues for selected UGT and SULT forms, they often exhibit distinct tissue-specific expression patterns and substrate affinities [37,38].

Therefore, while this research provides new insights into how specific metabolic conditions affect phase II drug-metabolising enzymes, these results should be interpreted with caution. They highlight the need for further validation at the protein and functional level in models that may more fully encompass the complexity of human MetS.

## 5. Conclusions

Despite the inherent limitations of mRNA-based analyses, this study identified pronounced, model-specific differences in hepatic and intestinal expression of phase II drug-metabolising enzymes across non-obese rat models of metabolic syndrome. In hypertriglyceridaemic HHTg rats, hepatic suppression of selected UGT isoforms, including loss of detectable Ugt2b transcripts, was accompanied by compensatory induction of sulfotransferases and increased intestinal UGT expression. In contrast, the hypertensive SHR model displayed a distinct hepatic adaptive pattern characterised by induction of multiple phase II enzymes without evidence of impaired Ugt2b expression. Chronic CRP-driven inflammation resulted in divergent regulation between the liver and intestine, with suppression of hepatic UGT expression alongside a marked induction of intestinal glucuronidation. Oestrogen deprivation in ovariectomised rats further demonstrated tissue-specific modulation of phase II enzyme expression.

These findings indicate that alterations in phase II metabolism are strongly dependent on the underlying pathophysiological context and tissue compartment. Such differences may influence drug disposition, first-pass metabolism and susceptibility to drug–drug interactions in preclinical models of cardiometabolic disease. However, confirmation at the protein and functional levels will be required to establish the translational relevance of these observations.

## Data Availability

Data supporting the findings of this study are available upon request from the corresponding author.

## Supplementary Materials

The following supporting information can be downloaded at: https://www.mdpi.com/article/10.3390/biomedicines14061206/s1, Table S1: List of primers used for real-time RT-qPCR.
