# Supplementary material for: Comparative Analysis of Selected UGT and SULT mRNA Expression in Non-Obese Rat Models of Metabolic Syndrome
Source: Biomedicines. 2026 May 27;14(6):1206. doi: 10.3390/biomedicines14061206 (PMC13296433; doi:10.3390/biomedicines14061206)
Supplement: Supplementary file 1 [file biomedicines-14-01206-s001.zip › biomedicines-4288530-supplementary.pdf]

Supplementary table S1: List of primers used for real-time RT-qPCR

Primer name	Batch number
Ugt1a1	Rn00754947_m1
Ugt2b7	Rn00821928_m1
Ugt1a6	Rn00561171_m1
Ugt1a9	Rn06299012_g1
Ugt1a8	Rn01764956_m1
Ugt2b2	Rn02349652_m1
Sult1a1	Rn01510633_m1
Sult1b1	Rn00673872_m1
Sult1e1	Rn00820646_g1
Sult2a1	Rn06220013_m1
Hprt1	Rn01527840_m1
